# Reading in English as a Foreign Language by Spanish Children With Dyslexia

**DOI:** 10.3389/fpsyg.2020.00019

**Published:** 2020-02-14

**Authors:** Paz Suárez-Coalla, Cristina Martínez-García, Andrés Carnota

**Affiliations:** Department of Psychology, University of Oviedo, Oviedo, Spain

**Keywords:** English as a foreign language, dyslexia, reading, Spanish, children

## Abstract

It has been reported that children with dyslexia have difficulties with learning a second language. The English alphabetic code is opaque, and it has been stated that deep orthographies cause important problems in children with dyslexia. Considering the strong differences between the Spanish and English orthographic systems, we predicted English reading problems in Spanish-speaking children with dyslexia. The current study focused on English as a foreign language in a group of 22 Spanish children with dyslexia (8–12 year olds), compared to a control group matched for age, gender, grade, and socioeconomic status. The objective was to identify the main difficulties that Spanish-speaking children with dyslexia demonstrate during English reading, to develop specific teaching programs. Participants were given four tasks related to reading: discrimination of phonemes, visual lexical decision, reading aloud, and oral vs. written semantic classification. The results suggest that children with dyslexia demonstrate problems in using English grapheme–phoneme rules, forcing them to employ a lexical strategy to read English words. However, they also showed difficulties in developing orthographic representations of words. Finally, they also exhibited problems with oral language, demonstrating difficulties accessing semantic information from an auditory presentation.

## Introduction

### Developmental Dyslexia

Developmental dyslexia is considered a neurobiological condition characterized by specific and pronounced difficulty in reading and writing acquisition. This condition results in persistent accuracy and speed deficits in both reading and writing competencies (Grainger et al., 2003; Lyon et al., 2003; Suárez-Coalla and Cuetos, 2012, 2015; Afonso et al., 2019). The origin of literacy acquisition problems has been repeatedly attributed to deficits in phonological processing or the ability to identify and manipulate speech sounds (Goswami and Bryant, 1990; Stanovich and Siegel, 1994; Serrano and Defior, 2008). Moreover, recent studies suggest that the phonological deficit could be partially caused by certain subtle disorders in sound perception, preventing children with dyslexia from developing good phonological representation (Goswami, 2002; Boets et al., 2006; Beattie and Manis, 2012; Cuetos et al., 2018). Consequently, disorders in sound perception could determine phonological awareness and literacy acquisition, which could cause more pronounced and profound problems for foreign language (FL) learning.

### Reading Acquisition

To achieve reading competence in the first/native language (L1), it is necessary to acquire the grapheme–phoneme (G–P) conversion rules, in addition to developing orthographic representations of intermediate units (groups of graphemes) and words. The development of orthographic representations of words is particularly pertinent in deep orthographic systems. It is well-recognized that the first step in learning to read is to learn the alphabetic code (Ehri, 1987, 1998; Share, 1995). This knowledge of G–P correspondences is critical, as it permits people to decode the written language. However, this knowledge is not sufficient in and of itself to constitute fluent reading skills. To develop reading fluency, we also need to store orthographic representations of words. This facilitates direct, smooth, and fast reading, without having to convert each grapheme into its corresponding phoneme. According to the self-teaching hypothesis, and supported by many studies, the accurate and repeated decoding of words facilitates the storing of the orthographic representation of those words in memory (Ehri and Roberts, 1979; Reitsma, 1989; Share, 1999; Cunningham, 2006; Kyte and Johnson, 2006; Maloney et al., 2009). In this sense, the acquisition and automation of the alphabetic code is crucial to obtaining an appropriate and robust orthographic representation of new words. However, the characteristics of the orthographic system seem to determine the evolution of the reading strategies. In deep orthographic systems, like English, G–P irregularities seem to force (from an early age) the development of orthographic representations of intermediate units (i.e. rhymes, syllables, and morphemes) and words (Wang et al., 2012). By contrast, in transparent orthographic systems, like Spanish or Italian, the G–P correspondence rules are very easy to learn, and children decode them accurately from the outset (Seymour et al., 2003; Cuetos and Suárez-Coalla, 2009). Nevertheless, even in transparent orthographies, children also develop representations for intermediate units (Burani et al., 2002; Cuetos and Suárez-Coalla, 2009), and for whole words (Suárez-Coalla et al., 2016). It has been reported that several variables modulate orthographic storing, including, for example, syllable structure, context-dependent graphemes, and phonological or semantic knowledge of new words (Bowey, 1995; Walley et al., 2003; Ricketts et al., 2007; Wang et al., 2013; Álvarez-Cañizo et al., 2018). The development of reading fluency therefore depends on many variables.

Regarding dyslexia, studies about reading difficulties support the idea that children with dyslexia demonstrate problems in learning the alphabetic code and creating orthographic representations of words. Evidently, if they do not successfully learn the alphabetic code – because of difficulties in phonological processing – and make mistakes when reading words, they will have problems storing correct representations (Bruck, 1992; Vellutino et al., 2004). It has been reported that children with dyslexia remain inaccurate and slow after a significant number of decoding opportunities (Hogaboam and Perfetti, 1978; Reitsma, 1983; Manis, 1985; Ehri and Saltmarsh, 1995; Cao et al., 2006; Martens and de Jong, 2008; Clements-Stephens et al., 2012; Suárez-Coalla et al., 2014b). These difficulties seem to lead to the use of a sublexical reading strategy. Children without reading difficulties, by contrast, use lexical reading strategies from an early age (Suárez-Coalla and Cuetos, 2012; Davies et al., 2013).

Furthermore, cross-linguistic studies have reported that the reading performance of people with dyslexia varies depending on the orthographic system, resulting in diverse behavioral manifestations, in spite of dyslexia’s common neurological origin. In particular, as a consequence of orthographic depth, it has been noted that dyslexic reading accuracy problems are more pronounced in deep orthographies (e.g. English) than in shallow ones (e.g. Spanish or Italian) (Wimmer, 1993; Wimmer and Goswami, 1994; Ziegler and Goswami, 2005).

Reading slowness constitutes the main marker of dyslexia in shallow orthographies (De Jong and van der Leij, 2002; Constantinidou and Stainthorp, 2009; Suárez-Coalla and Cuetos, 2012). In Spanish, slowness seems to be a consequence of using a sublexical strategy for reading aloud and the lack of mastery of the G–P rules. Specifically, Spanish-speaking children with dyslexia show a significant effect based on the length of the stimuli (a marker of a sublexical strategy) and continue to show the length effect after repeated exposure to words (Suárez-Coalla et al., 2014a, b; Martínez-García et al., 2019). It is thought that this persistent length effect is an indicator of the absence of orthographic information (Suárez-Coalla et al., 2014a, b; Martínez-García et al., 2019).

### Reading in English as a Foreign Language

Children all over the world learn English as an FL (EFL) at an early age (Bonifacci et al., 2017). Schools try to prepare children for a global society, and the language barrier constitutes a challenge to children with dyslexia. They must learn two different – sometimes widely divergent – alphabetic codes (e.g. English vs. Spanish). Spanish has a highly transparent orthography, with high correspondence between graphemes and phonemes (Seymour et al., 2003). Spanish speakers pronounce the majority of graphemes without variation, except for three consonants (c, g, and r). There are certain rules, however, which regulate the pronunciation of these consonants in relation to accompanying vowels and their position in a word. Moreover, Spanish has five double-letter graphemes (ll, rr, ch, gu, and qu), which only appear at the beginning of the syllable. In addition, there are only five vowels (a, e, i, o, and u), and their pronunciation does not vary. In general, the method of reading instruction in Spanish is phonetic–syllabic: children learn single letters and their sounds and then combine letters to read syllables and words. Therefore, because of the consistency of Spanish orthography, children without problems achieve reading accuracy very early on (i.e. during the first year of exposure to reading) (Seymour et al., 2003). However, this is not the case for children with dyslexia (Davies et al., 2007; Suárez-Coalla and Cuetos, 2012).

By contrast, English orthography is more irregular. In the English alphabet, there are 26 letters (21 consonants and 5 vowels, 6 if we consider “y,” also a vowel when it is the only “vowel” in a word, e.g. “sky”), but there are more than 40 consonant and vowel sounds. In some words (e.g. “best”), the number of letters and sounds is the same (four letters and four sounds). In other words, however (e.g. “green”), the number of letters and sounds is different (five letters and four sounds). In addition, some words have the same pronunciation but different spellings (e.g. “know” vs. “no”), and some have the same spellings but different pronunciation (e.g. “read:” infinitive vs. past tense) (Marks, 2007). These irregularities make it difficult to read EFL, particularly for children with dyslexia.

In Spain, children begin to be informally exposed to English from the beginning of preschool, when they are around 3 years old. However, English is introduced in a more formal and academically rigorous way in Year 1 of Primary School, at the age of 6. Currently, children receive EFL lessons for approximately 4 h/week. In addition, increasing amounts of bilingual education are being introduced in Spain, with ∼50% of subjects being taught in the English language. To teach reading in English, instructors mainly use a global method – introducing meaning, pronunciation, and spelling at the same time. This constitutes a significant challenge.

It is well-known that English reading causes particular difficulties for children with dyslexia (Nijakowska, 2010), and in Spain, these difficulties are often noted by parents and teachers. However, these difficulties are rarely assessed by clinicians and speech therapists, probably due to the absence of formal EFL testing, as well as the absence of specific training in EFL testing, and the traditional priority given to L1, as mentioned by Helland and Kaasa (2005) in the Norwegian context. Therefore, research in this field is critically necessary to develop an understanding of how Spanish children with dyslexia tackle reading in EFL.

Pioneering and influential studies about FL learning difficulties have advanced the Linguistic Coding Deficits Hypothesis (LCDH) (Sparks et al., 1999, 2012). This asserts that FL acquisition is related to phonological, orthographic, syntactic, and semantic skills in L1. The LCDH suggests that FL learning is built on L1 skills. Therefore, the strength of the L1 codes determines the student’s future success in FL learning. The assumptions derived from the LCDH have attracted the attention of multiple researchers. Specifically, it has been argued that people with reading problems in L1 will be prone to reading problems in an FL – that is, early problems with phonological and orthographic processes in L1 will be transferred to the FL (Chodkiewicz, 1986; Durgunoglu et al., 1993; Cisero and Royer, 1995; Da Fontoura and Siegel, 1995; Geva et al., 1997; Comeau et al., 1999; Dufva and Voeten, 1999; August et al., 2001; Kahn-Horwitz et al., 2006).

Accordingly, studies addressing reading in EFL (China, Italy, Norway, and Poland, etc.) have reported worse English reading performance in people with dyslexia than in typical readers, regardless of the characteristics of the L1 orthographic system (Chinese: Ho and Fong, 2005; Chung and Ho, 2010; Hebrew: Oren and Breznitz, 2005; Italian: Palladino et al., 2013; Norwegian: Helland and Kaasa, 2005; Polish: Lockiewicz and Jaskulska, 2016).

Lockiewicz and Jaskulska (2016) performed a study with Polish adolescents with dyslexia (aged 16–18), in which they were asked to read English words and pseudo-words. Significant differences were found between typical readers and adolescents with dyslexia. Students with dyslexia showed less accuracy and a slower reading speed than the control group, in both English words and pseudo-words. In another study, Palladino et al. (2013) compared Italian-speaking children with and without dyslexia (aged 12–14). In this study, children were also asked to read English words and pseudo-words. The Italian children with dyslexia showed poor reading of English words; however, contrary to the results reported by Lockiewicz and Jaskulska (2016), they seemed to manage English G–P rules because they showed a high level of accuracy when reading pseudo-words. Considering Norwegian students (aged 12), Helland and Kaasa (2005) found significant differences between groups in literacy tasks, who were tested on spelling, translation, and reading skills. In the context of the Chinese language, where the script differs significantly from that of English, Ho and Fong (2005) found that primary school children with dyslexia performed significantly worse than the control group in several English measures (vocabulary, reading, and phonological processing tasks). In addition, they found that phonological skills correlated with English reading.

Primarily, results have suggested that reading problems in L1 are a predictor of reading difficulties in EFL, probably due to a common cause. However, Miller-Guron and Lundberg (2000) reported surprising results. They found that some Swedish adults with dyslexia demonstrated a preference for reading in English, instead of Swedish (L1), even though Swedish orthography is more transparent than that of English. This phenomenon was termed the “dyslexic preference for English reading,” and it was believed to be a consequence of different factors, including age, and EFL exposure (mass media, literature, etc.). It is also believed to be related to a preference for larger orthographic segments, due to their inherent challenges with G–P decoding. These results could be modulated by other variables, but they are not generalizable. In this sense, it is interesting to continue researching about reading performance and strategies in EFL, especially in populations with different L1 orthographic systems (and different sociocultural contexts).

Regarding differences between L1 and English orthographic systems, it is reasonable to anticipate certain difficulties when learning two different alphabetic codes. For example, it must be considered that the complex graphemes of English (e.g. ea, ph, and ow – which do not exist in Spanish) could pose a difficulty to Spanish children. This graphemic complexity effect has been found in French children when reading English words (Commissaire, 2012), suggesting that the identification of complex graphemes competes with the identification of simple graphemes. On the other hand, the phonological representation of the words seems to be activated automatically during the visual recognition of words in bilingual individuals (Dijkstra and van Heuven, 2002); the same holds true for the grapheme–phoneme rules of both languages (Goswami et al., 2001; Jared and Kroll, 2001; Van Wijnendaele and Brysbaert, 2002). This finding suggests that differences in the orthographic systems could cause interferences to readers in EFL, especially when L1 is a transparent orthography (like Spanish). However, the level of activation could depend on the individual’s fluency and experience with languages (Jared and Kroll, 2001). It has been proven, however, that university students who are learners of EFL are sensitive to the morphological structure of English words, indicating that they are able to recode the written word into different grain sizes of psycholinguistic units (Casalis et al., 2015). Moreover, the “grain size accommodation” hypothesis has recently suggested that learning to read in consistent and inconsistent orthographies concomitantly is in fact advantageous to readers (Lallier and Carreiras, 2017). Readers in this context seem to increase their use of phonological strategies in opaque orthographies and lexical processing in transparent orthographies.

To our knowledge, there are no studies about reading in English by Spanish-speaking children with dyslexia. Taking into account previous results, as well as the difficulties reported by teachers and parents, it can be expected that dyslexic Spanish children will have problems with reading in EFL.

The aim of this study was to describe specific difficulties of – and reading strategies used by – Spanish children with dyslexia in EFL reading, compared to typical Spanish readers. Specifically, we tried to determine whether Spanish children with dyslexia were able to use some English G–P rules to read unfamiliar words or, alternatively, whether they had difficulties managing English regularities. We also tried to determine if they had developed the orthographic representations of words or, instead, whether there was some kind of phonological and/or cross-linguistic interference (i.e. if they activate the Spanish phonology when reading in EFL). Furthermore, we intend to evaluate whether problems with the discrimination of phonemes were also noticeable in this population, as it has been argued that auditory deficits exist in people with dyslexia, which probably affect phonological representations and therefore English learning. To achieve our goals, four tasks were performed: discrimination of phonemes (same–different), visual lexical decision-making, reading aloud, and oral vs. written semantic categorization. Participants were native Spanish speakers with and without dyslexia, from 8 to 12 years old, who studied EFL as a compulsory subject at school. We assumed that children with dyslexia would show worse performance in all tasks, with more mistakes and longer reaction times (RTs) than children without dyslexia.

In short, we are seeking to address the following issues related to EFL reading:

- Do children with dyslexia have representations of English phonemes and the ability to discriminate among them?
- Do children with dyslexia know the G–P conversion rules of English and use them to read unknown words?
- Do children with dyslexia developed robust orthographic representations of English words?
- Are children with dyslexia able to access semantic information from oral and/or written presentations? Do they demonstrate differences depending on the form of presentation of the stimuli?

## Methodology

### Participants

A total of 44 children (24 boys and 20 girls) between 8 and 12 years of age (*M* = 10 years, 8 months, *SD* = 0.8) participated in the study. Half of the participants had dyslexia (DYS), and half were typical readers (CON). Both groups were matched by age, gender, grade, and socioeconomic status. All the participants were native Spanish speakers and had no known motor, cognitive, or perceptual disorders.

Participants without dyslexia were recruited from several primary schools in Asturias (Spain). The children with dyslexia were recruited from the Association of Dyslexia and certain Speech Therapy Centers of Asturias (Spain). Children with dyslexia had previously received the diagnosis of dyslexia, had an intelligence quotient (IQ) of 85 or higher (*M* = 109; *SD* = 7.58), according to the Wechsler Intelligence Scale for Children (Wechsler, 2001), and had shown a low phonological awareness performance (in a phoneme omission task created by the authors of the study). The average score in the phonological awareness task was *M* = 6.67 (out of 10), *SD* = 1.70. The average score for the typical readers was *M* = 9.40, *SD* = 0.87.

Before performing the experimental tasks, a reading battery (PROLEC-R, Cuetos et al., 2007, or PROLEC-SE, Ramos and Cuetos, 2005) was administered to all participants, to confirm the diagnosis of reading difficulties. PROLEC-R and PROLEC-SE yield scores (accuracy and total reading times) for words and pseudo-words. The section of words consists of 40 Spanish words (high and low frequency, short, and long words). The pseudo-words section includes 40 pseudo-words, half of which were short and half of which were long. Children were included in the DYS group if both accuracy and reading speed scored 1.5–2 standard deviations below the age mean, according to age norms provided by PROLEC-R or PROLEC-SE. Meanwhile, children were included in the CON group when they had an age-appropriate score in both sections. Means, standard deviations, and *p* values for scores obtained in reading assessment tests are provided in Table 1.

**TABLE 1:** Means, standard deviations and *p*-values for scores obtained in reading assessment tests.

	**DYS mean (*SD*)**	**CON mean (*SD*)**	***p*-value**
Age (years)	10.9 (0.9)	10.7 (0.8)	*p* = 0.67

**Reading**			
**Words**			
Accuracy (out of 40)	35.83 (1.86)	39.76 (0.59)	*p* < 0.001
Speed (s)	62.78 (28.40)	30.22 (13.35)	*p* < 0.001
**Pseudowords**			
Accuracy (out of 40)	33.50 (2.85)	38.11 (0.81)	*p* < 0.001
Speed (s)	80.06 (18.84)	42.25 (9.35)	*p* < 0.001

### Materials and Methods

Four tasks were performed: discrimination of phonemes (same–different), visual lexical decision-making, reading aloud, and oral vs. written semantic categorization. Each task lasted ∼5 min.

#### Discrimination of Phonemes: Same–Different

The relationship between phonological processing and reading acquisition is well-known (Wagner and Torgesen, 1987;Adams, 1990; Scanlon et al., 2000; Snowling, 2000). In line with the definition provided by Catts et al. (1999), phonological processing includes the perception, storage, recovery, and manipulation of language sounds. The ability to manipulate speech units requires an important level of awareness that words are formed by sublexical segments (i.e. discrete sounds that can be recombined). Different tasks (omission and identification of phonemes, rhyming judgments, word segmentation, and discrimination of phonemes) are used to assess phonological awareness and its relation to reading.

In English, there are more vowel phonemes than in Spanish, some of which have subtle differences that are very difficult for Spanish people to discriminate (e.g. the sound/I/as in “sit” vs. the sound/i:/as in “seat”). This could pose a problem to Spanish children with phonological processing deficits, such as children with dyslexia (Elbro, 1996; Snowling, 2000). By contrast, it has also been suggested that children with dyslexia – who have problems acquiring the phonological categories of L1 – retain sensitivity to universal phonetic boundaries, which are lost in typical phonological acquisition (Serniclaes et al., 2004; Soroli et al., 2010). In this sense, this task aimed to ascertain whether children with dyslexia have problems discriminating English vowel phonemes.

A total of 36 English monosyllabic words were selected. From the selected stimuli, 12 pairs, including pairs featuring the same word, were formed (e.g. hot–hot). From the remaining stimuli, 12 pairs were created, including two different words which only differed in one phoneme (e.g. sheep–ship). In addition, four trials were included at the beginning as practice, to familiarize children with the task.

Participants were orally presented with pairs, and they had to decide whether the pairs contained the same word or different words. They were told that they were going to hear two stimuli, and they were asked to decide, as quickly and accurately as possible, whether they were the same or different by pressing the appropriate key. One key had a green sticker placed on top, which was used to indicate that the words were the same, and another key, which a red sticker placed on top, was used to indicate that the words were different.

To obtain the auditory stimuli, words were recorded with a Zoom H4N recorder and Audix Ht2-P Plantronics microphones. Subsequently, the stimuli were edited with Praat software (Boersma and Weenink, 2019). The experimental task was run on an HP Mini laptop, with the DMDX program (Forster and Forster, 2003). The trial started with a warning tone and an asterisk on the screen, followed by the two words. A silent interval of 500 ms was placed between the two stimuli. Timing started from the onset of the second stimulus. The type of response (correct or incorrect) and RTs were recorded as data. Cronbach’s alpha was 0.55.

#### Reading Aloud

According to dual-process models, reading may be conducted through two different processing routes. The sublexical route uses knowledge about the alphabetical code: the G–P rules of the language. Alternatively, the lexical route makes use of the orthographic representations of words to lexically access their phonological representations (Coltheart and Rastle, 1994; Coltheart et al., 2001). When you have to read an unknown word, you do not have a pre-existing orthographic representation of it. Therefore, you have to use the alphabetical code or use some kind of analogy to obtain the correct pronunciation. With this task, we tried to determine if the children with dyslexia were able to read infrequent and unfamiliar words based on the knowledge of certain G–P conversion rules of the English orthographic system. When reading aloud regular words, it is not necessary to know the orthographic representation of the word or the word meanings. This task could therefore inform us about the ability of dyslexic children to manage English G–P rules.

A total of 24 words were selected. Half of them were high-frequency (HF) words (*M* = 63,198, *SD* = 86,807) and were considered familiar to children, as they were drawn from their English schoolbook [e.g. “t**a**ble” (’te**I**b**ə**l)]. The other half of the words were low-frequency (LF) words (*M* = 1,388, *SD* = 3,240), previously unknown to the children [e.g. “g**a**ble” (’g**eI**bəl)]. The lexical frequency was obtained from the Hyperspace Analog to Language (HAL) frequency norms (Lund and Burgess, 1996). These frequency norms were based on the HAL corpus, which consists of ∼131 million words gathered across 3,000 Usenet newsgroups during February 1995, cited in *The English Lexicon Project* (Balota et al., 2007). These unknown words were orthographic and phonological neighbors to the known ones, since the two words differed only in a consonant phoneme. The vowel phoneme remained the same (in terms of spelling and pronunciation).

From these words, we created two lists of words matched on frequency and pronunciation, so that children received six known and six unknown words. Each word was presented visually (20-point Arial font) to participants for 4,000 ms. They were asked to read the word aloud as quickly and accurately as possible. RTs were considered – that is, the duration between the onset of the target on the screen and the time when the participants started to articulated the word.

The experimental task was run on an HP Mini laptop, and the responses were recorded in.WAV files with the DMDX program (Forster and Forster, 2003). A trial started with a warning tone and an asterisk on the screen, followed by the word to be read. The sound spectrograms of the recorded responses were analyzed using the CheckVocal application (Protopapas, 2007) to extract accuracy and RTs. Mistakes (self-corrections, substitutions, and regressions) and omitted responses were excluded. Cronbach’s alpha was 0.88.

#### Lexical Decision-Making Task

To perform a visual lexical decision-making task, it is necessary to have developed a robust orthographic representation, especially when it comes to irregular words. On the other hand, it has been reported that the L1 phonology is activated even when an individual is reading an FL. In this sense, the visual decision task has been used to ascertain the influence of L1 in FL word recognition (Elston-Güttler et al., 2005). It is relevant when L1 and FL phonemes differ considerably, as they do in Spanish and English. The objective of this task was to ascertain if children with dyslexia had developed orthographic representations of English words or, alternatively, if they were affected by phonological cross-linguistic interferences.

In this task, the participants had to recognize and decide if a visually presented letter sequence constituted a real word. A total of 32 stimuli were selected, manipulating lexical frequency and length. For the short stimuli, the mean length was 3.75 (*SD* = 0.43, three to four letters), and for the long stimuli, the mean length was 7.55 (*SD* = 0.96, six to nine letters). Regarding the frequency values of words, the mean for the HF words was 176,051 (*SD* = 77,138), and for the LF words, the mean was 6,988 (*SD* = 3,470). The frequency values were obtained from the HAL frequency norms (Lund and Burgess, 1996, cited in Balota et al., 2007). Sixteen stimuli were presented in their correct spellings (e.g. “cake”), with eight short stimuli and eight long stimuli. Half of these were HF, and half of these were LF. Sixteen stimuli were presented with incorrect spellings, with phonologically plausible errors according to the phonological representation and Spanish pronunciation (i.e. pseudo-homophones whose transcriptions followed Spanish phonological rules, e.g. “yiar” instead of “year”). To respond, participants had to press – as quickly as possible – a key on the computer keyboard (the green key if the letter sequence constituted a real word, and the red key if not). Stimuli were presented in lowercase letters (Arial, 20-point font) at the center of the screen (black on white) using DMDX software (Forster and Forster, 2003). Each stimulus remained for 4,000 ms on the screen, replaced by an asterisk as a fixation point for 500 ms, followed by a blank screen for another 500 ms. In addition, before starting, four practice trials were run to familiarize the participants with the task. The types of responses and the RTs were recorded as data. RTs were considered to be the duration between the onset of the target on the screen and the time at which the participant pressed the key. Cronbach’s alpha was 0.83.

#### Oral and Written Semantic Classification Task

The final objective of reading is to access semantic information, which is a step toward text reading comprehension. From a logical point of view, children with dyslexia should have more difficulties in obtaining semantic information from the written word than from the orally presented words, as their principal problem is in reading. However, considering the phonological difficulties of children with dyslexia and differences between Spanish and English phonology, it would also be consistent to argue that they have inaccurate phonological-auditory representations that will make oral recognition difficult. Using this task, we wanted to ascertain whether children with dyslexia exhibited similar performance when accessing semantic information from an auditory stimulus and from a written one.

We included two modalities: oral and written presentations. For each modality, 3 semantic categories and 24 stimuli (8 per category) were selected. For the written modality, we considered the following categories: animals, body parts, and professions. For the oral modality, we considered food, clothes, and household objects. The same categories were not used in both versions (oral and written) to avoid a facilitating effect by presenting the same category twice. These kinds of categories were chosen because they receive the same levels of attention in the English textbooks for the third and fourth grades of primary education in Spain. In addition, the selected items for each category appear as part of the vocabulary in the cited English textbooks. The stimuli of the different semantic categories are matched in the number of letters (*M* = 4.9, *SD* = 1.2), phonemes *(M* = 3.8, *SD* = 0.9), syllables *(M* = 1.3, *SD* = 0.47), and lexical frequency (*M* = 10,947, *SD* = 7,715), according to the HAL frequency norms (Lund and Burgess, 1996, cited in Balota et al., 2007).

**FIGURE 1:**
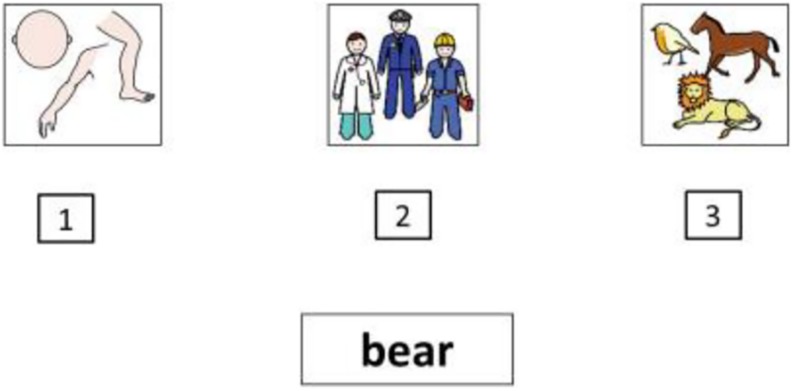
Example of written semantic categorization task.

Participants received the stimulus (either written on the screen or orally by headphones), and they had to classify it as belonging to one of the three categories considered in the modality. To classify the stimuli, three pictures and one number (1, 2, and 3), associated with each category, were presented on the screen, and participants had to select the correct picture by pressing the assigned number on the keyboard (see Figure 1). The auditory stimuli were recorded by a 9-year-old bilingual girl with a Zoom H4N recorder and Audix Ht2-P Plantronic microphone. Subsequently, the stimuli were edited with Praat software (Boersma and Weenink, 2019). The experimental task was run on an HP Mini laptop using the DMDX program (Forster and Forster, 2003). Cronbach’s alpha for the written version was 0.86, and Cronbach’s alpha for the oral version was 0.72.

The research design and protocol were approved by the Ethics Committee for Research of the Principality of Asturias, Spain. The study was developed in accordance with the Declaration of Helsinki and the principles of the Spanish Law of Personal Data Protection (15/1999 and 3/2018). A written informed parental consent was received for all participants, authorizing the students to take part in the experiment (Figure 1).

## Results

For RTs, ANOVAs were performed with mixed-effects analyses (Baayen, 2008) using R-software (R Core Team, 2016), with participants and items as random-effect variables. As fixed factors, we considered the group factor (DYS vs. CON), as well as different factors according to the task (word frequency, length, spelling type, presentation type, or type of stimuli). Stepwise model comparisons were conducted, from the most complex to the simplest model and the one with the most complex adjustment but the smallest Bayesian information criterion and the significant χ^2^ test for the log-likelihood was retained (Schwarz, 1978). *F* values from the ANOVAs of type III, with the Satterthwaite approximation for degrees of freedom, were reported for fixed-effects variables. If interactions were significant, *t* tests were performed, and the *p* values were adjusted via the Holm–Bonferroni method. For the analysis of errors, we used a generalized mixed-effect model with a binomial distribution. A *p* < 0.05 was adopted as a level of significance.

### Discrimination of Phonemes: Same–Different

In this task, we analyzed RTs and accuracy, considering group (CON vs. DYS) and stimuli type (same vs. different) as fixed-effects variables. For the analysis of RTs, we found type of stimuli effect [*F*(1,17.578) = 5.1471, *p* < 0.05), where RTs were longer for the different-stimuli pairs than for the same-stimuli pairs (estimate = 142, *SE* = 62.4; effect size = 0.72).

We found the same effect when accuracy was considered, with differences between same and different stimuli (*p* < 0.01; estimate = 1.72, *SE* = 0.55; OR = 4.95; CI = 1.47–16.63; effect-size = 0.43), as they showed a higher probability of making mistakes in different-stimuli pairs than in same-stimuli pairs. The group effect was not significant, suggesting that children with dyslexia do not have specific problems discriminating English phonemes or, alternatively, that they performed similarly to the CON group.

### Reading Aloud

In the reading aloud task, we analyzed RTs and accuracy. The fixed factors were group (CON vs. DYS) and lexical frequency (HF vs. LF). We identified a group effect [*F*(1,39.173) = 9.794, *p* < 0.01], as RTs were longer in the DYS group than in the CON group (estimate = 273, *SE* = 91.3; effect size = 0.46). We also identified a lexical frequency effect [*F*(1,19.911) = 24.933, *p* < 0.001], as RTs were longer in LF words than in HF words (estimate = 261, *SE* = 55.3; effect size = 0.47).

Similar results were found when accuracy was considered (group effect: *p* < 0.001, estimate = 2.5, *SE* = 0.57; OR = 0.08, CI = 0.02–0.25; effect size = 0.95; and lexical frequency effect: *p* < 0.001, estimate = 2.6, *SE* = 0.58; OR = 0.07, CI = 0.02–0.2; effect size = 0.96). These results indicated that the DYS group showed more mistakes than the CON group. Moreover, results were better for HF words than LF words, independently of the group.

### Lexical Decision-Making Task

In this task, we analyzed RTs and accuracy. The fixed factors were group (CON vs. DYS), length (short vs. long), lexical frequency (high vs. low), and spelling type (correct vs. incorrect).

We found a marginally significant group effect [*F*(1,39.98) = 3.389, *p* = 0.07, estimate = 228, *SE* = 124; effect size = 0.51], length effect [*F*(1,28.63) = 18.58, *p* < 0.001, estimate = 239, *SE* = 58.1; effect size = 0.52], and spelling type effect [*F*(1,29.59) = 6.23, *p* < 0.05, estimate = 131, *SE* = 52.4; effect size = 0.53). These results indicated that RTs were longer for DYS children than for CON children, for long as opposed to short stimuli, and incorrect as opposed to correct spelling stimuli.

**FIGURE 2:**
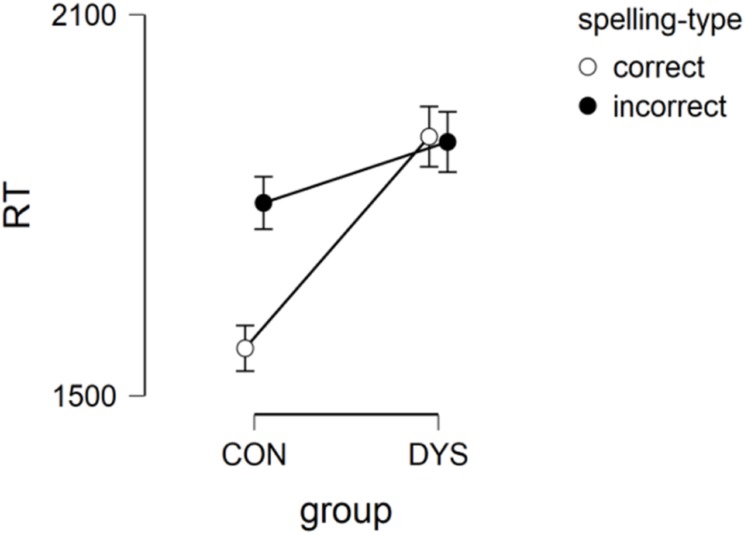
RT group by spelling type interaction in Lexical decision task.

We also found group × spelling type interaction [*F*(1,908.22) = 6.71, *p* < 0.01]. Pairwise comparison showed differences between correct and incorrect stimuli in the CON group [*p* < 0.01, *t* (49.2) = 3.744, estimate = 222.6, *SE* = 59.5; effect size = 0.50]. The difference between CON and DYS in the correct stimuli was marginally significant [*p* = 0.09, *t* (45.8) = 2.496, estimate = 319.5, *SE* = 128; effect size = 0.48). See Figure 2.

**FIGURE 3:**
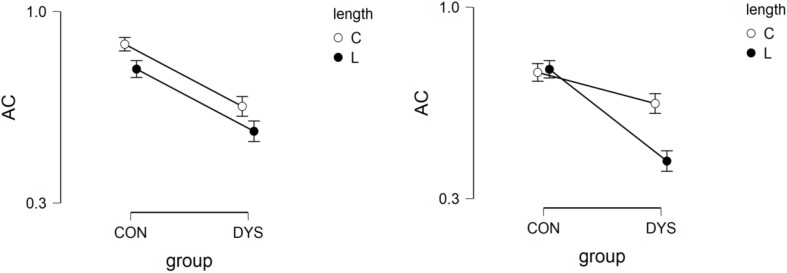
Accuracy in group by spelling type by length interaction in Lexical decision task (**left**: correct spelling, **right**: incorrect spelling).

In accuracy, we found a group effect (*p* < 0.001, estimate = 1.29, *SE* = 0.24; OR = 0.21, CI = 0.105–0.425; effect size = 0.93); length effect (*p* < 0.05, estimate = 0.516, *SE* = 0.21; OR = 0.49, CI = 0.23–1.04; effect size = 0.71); spelling type effect (*p* < 0.05, estimate = 0.40, *SE* = 0.21; OR = 0.40, CI = 0.19–0.85: effect size = 0.66); and group × length × spelling type interaction (*p* < 0.01). Pairwise comparison showed differences between CON and DYS in the short correct stimuli (*p* < 0.001, estimate = 1.55, *SE* = 0.36; effect size = 0.87); long correct stimuli (*p* < 0.01, estimate = 1.26, *SE* = 0.32; effect size = 0.91); and long incorrect stimuli (*p* < 0.001, estimate = 1.68, *SE* = 0.32; effect size = 0.89). In addition, differences between short and long incorrect stimuli in the DYS group were marginally significant (*p* = 0.08, estimate = 0.97, *SE* = 0.33; effect size = 0.61). See Figure 3.

Results suggested that the CON group had more orthographic representations than the DYS group.

### Oral and Written Semantic Classification Task

**FIGURE 4:**
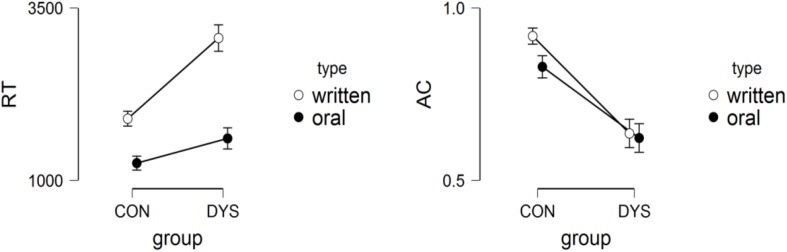
Reaction Times **(left)** and Accuracy **(right)** in group by presentation type interaction in semantic categorization task.

We considered group (CON vs. DYS) and presentation type (oral vs. written) as fixed factors. For RTs, we found a group effect [*F*(1,42.53) = 12.83, *p* < 0.001, estimate = 830, *SE* = 232; effect size = 0.38]; presentation type [*F*(1,1,528.33) = 309.541, *p* < 0.001, estimate = 1039, *SE* = 59.1; effect size = 0.74]; and group × presentation type interaction [*F*(1,1,523.13) = 55.223, *p* < 0.001]. Pairwise comparison showed differences between groups only in written presentation (*p* < 0.001, estimate = 1,266, *SE* = 238.7; effect size = 0.36), and differences between presentation type in both DYS (*p* < 0.001, estimate = 1,475, *SE* = 90.2; effect size = 0.32) and CON (*p* < 0.001, estimate = 603, *SE* = 75.7; effect size = 0.68) groups. Children with dyslexia showed worse performance in the written version than typical readers. See Figure 4.

In accuracy, we found a group effect (*p* < 0.001, estimate = 1.73, *SE* = 0.22, OR = 0.11, CI = 0.06–0.19; effect size = 0.92); presentation type (*p* < 0.001, estimate = 0.51, *SE* = 0.12, OR = 0.38, CI = 0.26–0.58; effect size = 0.66); and group by presentation type interaction (*p* < 0.001). Pairwise comparison showed differences between groups in both oral presentation (*p* < 0.001, estimate = 1.29, *SE* = 0.24; effect size = 0.88) and written presentation (*p* < 0.001, estimate = 2.17, *SE* = 0.26; effect size = 0.94), but differences between presentation type only occurred in the CON group (*p* < 0.001, estimate = 0.95, *SE* = 0.21; effect size = 0.70), with more mistakes in the oral than in the written presentation.

## Discussion

The aim of this study was to identify specific difficulties of Spanish children with dyslexia when conducting English reading, compared to typical Spanish readers. Specifically, we tried to determine whether children with dyslexia know and use English G–P rules to read unfamiliar words or, alternatively, whether they have difficulties managing English regularities. We also tested whether they had orthographic representations of words or whether they suffered from any Spanish phonological interference. Finally, we evaluated if phonological discrimination problems were also visible in this population. To achieve our aims, four tasks were performed: discrimination of phonemes, visual lexical decision-making, reading aloud, and oral vs. written semantic categorization. Spanish children with and without dyslexia, ages 8–12, were tested.

The results suggest that Spanish children with dyslexia do not demonstrate specific problems discriminating English vowel phonemes. They performed in a similar way to children without dyslexia in terms of both RTs and accuracy. They produced better results in same-stimuli pairs compared to different-stimuli pairs. These results contradict hypotheses stating that the ability to discriminate phonemes could influence reading performance. It has been repeatedly reported that dyslexia is characterized by phonological problems, suggesting that impaired phonological or auditory processing is the origin of the reading disorder (Ahissar et al., 2000; Goswami, 2011; Peterson and Pennington, 2012). According to this view, it is possible that the stimuli or the task were not good enough to capture the repeatedly reported phonological problems in people with dyslexia. However, alternative explanations could also be offered. First, the absence of differences could be a consequence of the age of participants, as phonological processing improves with reading experience (Perfetti et al., 1987; Morais, 1991). In this sense, another, more demanding, task would be more informative about phonological difficulties. Finally, according to the typical phonetic boundaries acquisition in L1, it is possible that children with dyslexia benefit from their difficulty in acquiring phonetic boundaries in L1, retaining the sensitivity to universal phonetic boundaries (Serniclaes et al., 2004; Soroli et al., 2010). However, it should be noted that the reliability of the phonemes discrimination task was low, so results cannot be considered as sufficiently consistent.

On the other hand, DYS children showed worse performance than CON children in all other tasks (reading aloud, visual lexical decision-making, and semantic categorization).

Considering the reading aloud task, designed to determine whether DYS children use some English G–P rules, we observed that they made more mistakes and were slower than the CON group. A similar pattern was observed in Spanish reading, although the main problem in Spanish children with dyslexia is reading slowness (Suárez-Coalla and Cuetos, 2012). However, similar to typical readers, they showed a lexical frequency effect in accuracy and RTs, so they performed better in HF than in LF words. These data seem to suggest that Spanish children with dyslexia are not able to learn G–P rules, but that they have developed orthographic representations of English words. They preferably use a lexical instead of a sublexical strategy to read (although their performance was below that of the CON children), probably given the difficulty of learning the alphabetic code. When it comes to reading in Spanish, the transparency of the orthographic system facilitates the learning of the alphabetic code, but it is not the case for the English orthographic system. In this vein, it has been reported that deep orthographic systems force people to develop lexical reading strategies (Wang et al., 2012). Our results were not in line with those of Palladino et al. (2013), who found that Italian children with dyslexia (aged 13) were accurate in reading pseudo-words. Those results were interpreted by Palladino et al. (2013) as showing that the Italian children with dyslexia have the capacity to assimilate English pronunciation rules. In our case, we used very LF words, instead of pseudo-words, so that they could potentially benefit from the pronunciation rules in reading them. However, they did not seem to benefit from these pronunciation rules, suggesting that they were using lexical reading. This idea could be consistent with the dyslexic preference for English reading hypothesis (Miller-Guron and Lundberg, 2000). Miller-Guron and Lundberg (2000) reported that some Swedish adults with dyslexia (10 in their study) prefer to read in English than in Swedish. This preference seems to start at around the fourth grade, when Swedish children begin learning English at school. At this point in time, they have already experienced a failure with the Swedish alphabetical code. The authors hypothesized that some people with dyslexia, because of their problems with learning the alphabetic code and their knowledge about English inconsistencies, either prefer or force lexical reading. This interpretation was also suggested by Siegel et al. (1995), who argued that people with dyslexia try to compensate for the difficulty in mastering the phonemic strategy of 1:1 decoding, paying more attention to the orthographic form of English words.

To deepen our understanding of the strategies the children used during English reading, a visual lexical decision-making task was performed. In this task, real words and pseudo-homophones, whose transcriptions followed Spanish phonological rules, were included. With this task, we aimed to ascertain whether the children used a robust orthographic representation to recognize words or, alternatively, whether the Spanish phonological code affected the visual lexical decision-making task. The CON children had better performances than the DYS children, as they made fewer mistakes and were faster than the latter group. Moreover, the DYS children spent a similar amount of time on correct and incorrect stimuli, but the CON children were faster when reading real words. Finally, considering mistakes, we did not find differences between short and long stimuli in the CON group (both correct and incorrect). These data led us to conjecture that there were more robust orthographic representations in the CON group, expanding the previous data. We deduce that Spanish DYS children experience difficulties developing orthographic representations of words (Suárez-Coalla et al., 2014a, b), and they probably experience the influence of the Spanish phonological code more than typical readers.

As regards the semantic categorization task, our objective was to evaluate the possible differences between oral and written processing in DYS children. In the two previous tasks, it was not strictly necessary to know semantic information to complete the tasks. When reading aloud, the children could read words using G–P rules, and in the lexical decision-making task, they could recognize words using orthographic representations. However, in semantic categorization, they need to access the words’ meanings, allowing us to compare whether they obtained semantic information from oral and written presentations in the same way. The results indicated that DYS children showed worse performance in the written version than the CON group when RTs were considered, as they spent more time than the CON group on the written stimuli. In addition, they made more mistakes than the CON group in both the oral and written versions. It should be noted, however, that typical readers benefited from the written version in terms of accuracy, while children with dyslexia did not. Considering this result, we can conclude that the DYS group also had some difficulties with English oral processing, as they made more mistakes than the CON children in the oral version. This supports the argument that the DYS group has fewer phonological representations and a smaller vocabulary than the CON group. That concurs with the suggestion that there are different problems associated with dyslexia that affect language learning (Crombie, 2000). Concretely, we suggest that weakness in phonological processing, poor working memory, and slow speed of information processing could affect performance in oral semantic categorization in particular, and language learning in general.

To summarize, a series of experiments on reading in EFL and related tasks were performed with Spanish children with dyslexia. The results suggested that Spanish children with dyslexia demonstrate difficulties mastering English G–P rules, leading them to use a lexical strategy to read English words. However, they also demonstrated difficulties in developing orthographic representation of words, with significant consequences. Finally, the results suggested that they also show problems with oral language, demonstrating difficulties in deriving semantic information from auditory presentation.

Our results confirm previous studies on EFL reading in people with dyslexia. Previous studies have reported that English reading is a challenging task for this population. In addition, the results agree with the LCDH (Sparks et al., 1999, 2012) and subsequent studies specifically related to reading (Chodkiewicz, 1986; Durgunoglu et al., 1993; Cisero and Royer, 1995; Da Fontoura and Siegel, 1995; Geva et al., 1997; Comeau et al., 1999; Dufva and Voeten, 1999; August et al., 2001; Kahn-Horwitz et al., 2006). This supports the argument that reading problems in L1 transfer to reading in an FL, due to a common cause. In general, we confirm English reading differences between DYS and CON children, as has been previously reported (Chinese: Ho and Fong, 2005; Hebrew: Oren and Breznitz, 2005; Italian: Palladino et al., 2013; Norwegian: Helland and Kaasa, 2005; Polish: Lockiewicz and Jaskulska, 2016). However, it should be noted that our participants were younger than those of other studies (Italian: Palladino et al., 2013; Norwegian: Helland and Kaasa, 2005; Polish: Lockiewicz and Jaskulska, 2016), and the tasks were also different. In this vein, we found, contrary to the findings of Palladino et al. (2013), that Spanish children with dyslexia do not master the English G–P rules. According to Miller-Guron and Lundberg (2000), they seem to prefer a lexical strategy, but they also have problems with this strategy.

### Limitations

These outcomes help us to better understand how Spanish children with dyslexia address reading in EFL. However, more evidence is needed, as reading acquisition is a very complex process. Research in this field would allow us to design strategies to improve English language teaching and learning for children with dyslexia.

Our study has limitations that should be taken into account in the future. We tried to address some of the main difficulties that Spanish children with dyslexia show when they engage in EFL reading. We wanted to identify the reading strategies that Spanish children with dyslexia use. However, the size of the group was small considering the range of ages in the sample. Therefore, the results must be considered with caution. Furthermore, although there were no differences between the types of schools the children attended, other variables could have an important influence on our results (such as motivation, English vocabulary, and reading exposure, etc.). The findings support the argument that Spanish children with dyslexia demonstrate significant difficulties when reading in English. It is likely, however, that there are subgroups with different degrees of difficulties (perhaps affected by other variables, such as age, type of task, teaching methodology, English exposure, motivation to learn English, vocabulary level, etc.). In addition, it would be necessary to examine again the phonological awareness skills, as the task performed in this study was not sufficiently reliable. Finally, considering our results, other areas should be pursued, and a longitudinal study could contribute to greater knowledge about EFL acquisition in Spanish children with dyslexia.

## Data Availability Statement

The datasets generated for this study are available on request to the corresponding author.

## Ethics Statement

The studies involving human participants were reviewed and approved by the Ethics Committee for Research of the Principality of Asturias, Spain. Written informed consent to participate in this study was provided by the participants’ legal guardian/next of kin.

## Author Contributions

PS-C and AC contributed to the conception and design of the study. AC and CM-G collected and organized the database. PS-C performed the statistical analysis and wrote the manuscript. All authors read and approved the submitted version.

## Conflict of Interest

The authors declare that the research was conducted in the absence of any commercial or financial relationships that could be construed as a potential conflict of interest.
